# Association between delirium and grip strength in ICU patients for cardiac surgery (D-GRIP study)

**DOI:** 10.1186/s40981-023-00676-y

**Published:** 2023-11-25

**Authors:** Taichi Kotani, Satoki Inoue, Mitsuru Ida, Yusuke Naito, Masahiko Kawawguchi

**Affiliations:** 1 Department of Anesthesiology, Nara Medical University, 840 Shijo-cho Kashihara, Nara, 634-8521 Japan; 2 Department of Anesthesiology, Fukushima Medical University, Fukushima, Japan

To the Editor,

Postoperative delirium in cardiac intensive care units (ICUs) is associated with poor postoperative outcomes [1], and recent studies have investigated the effect of frailty on the incidence of postoperative delirium [2, 3]. Frailty, defined by criteria such as weight loss, exhaustion, low handgrip strength, slow walking speed, and low physical activity, is associated with a high incidence of postoperative delirium [4]. Low handgrip strength (< 28 kgf in males and < 18 kgf in females) in patients before cardiovascular surgery is associated with a higher incidence of postoperative delirium [5]. Therefore, we conducted this randomized controlled trial to test the hypothesis that preoperative grip strength training reduces the incidence of postoperative delirium. This single-center, single-anonymized, randomized controlled trial was approved by the Nara Medical University Institutional Review Board and registered at the UMIN Clinical Trials Registry (UMIN000041236). Patients aged > 60 years who underwent elective cardiac surgery at our hospital between August 8, 2020, and August 7, 2023, were included. Males and females with preoperative handgrip strengths of > 30 and > 20 kgf, respectively, were excluded.

Among 125 potential participants, 83 were excluded for high grip strength, and 29 refused to participate. The remaining 13 patients were randomly grouped, but one patient withdrew his consent midway, and three could not be assessed for postoperative delirium due to a consciousness disorder (Supplementary Fig. 1). Five and four participants were finally included in the intervention and control groups, respectively (Supplementary Table 1a, b). The intervention group performed grip strength training at home for at least 14 days preoperatively. Delirium was assessed thrice daily until ICU discharge using the confusion assessment method for the ICU [6]. The primary outcome was the difference between the incidence of postoperative delirium in the groups determined using Fisher’s exact probability test. We discontinued this study before the recruitment of the entire study population on August 7, 2023, because the participants were not likely to increase. The intervention group had a mean age of 75 years and included three males. The control group had a mean age of 75 years and included only males. The average grip strength of the intervention group increased from 23.6 kgf before the intervention to 28.8 kgf 1 day preoperatively. The control group had an average grip strength of 25.0 kgf 1 day preoperatively. The incidence of postoperative delirium was 40% and 75% for the intervention and control groups, respectively (Table 1).

**Table 1:** Postoperative outcomes in the intervention and control groups

	Intervention *n* = 5	Control *n* = 4	*p*-value
Postoperative delirium	2 (40)	3 (75)	0.34
Mortality at 30 days	0 (0)	0 (0)	Not applicable
Increased rate of handgrip strength (%)	22.5 ± 7.4	5.4 ± 7.5	0.016

Data are presented as mean ± SD or cases (%)

*SD* standard deviation

The reasons for the few participants include the following. First, several studies have reported that preoperative interventions should be restricted to patients with poor conditions, as characterized by frailty [7, 8]. However, only a few patients undergoing cardiac surgery met this criterion. Second, a common reason for the exclusion of participants was their apprehension about participating in this study. Preoperative anxiety may have contributed to their refusal to participate [9]. The Japanese or domestic cultural backgrounds also had effects.

Due to the large number of patients with high pre-intervention grip strength and the difficulty in obtaining consent, it was impossible to complete this study with the small number of patients we could include. While we discontinued this study due to the small sample, the potential role of training in improving grip strength and reducing postoperative delirium has been highlighted.

### Supplementary Information

**Additional file 1: Supplementary Figure 1.** CONSORT 2010 Flow Diagram.

**Additional file 2: Supplementary Table 1.** a Demographic characteristics of the participants. b Intraoperative and ICU data of the participants.

## Data Availability

Data supporting the findings of this study are available from the corresponding author upon request.

## Abbreviations

ICU: Intensive care unit
SD: Standard deviation
