# Supplementary material for: Association between delirium and grip strength in ICU patients for cardiac surgery (D-GRIP study)
Source: JA Clin Rep. 2023 Nov 25;9:81. doi: 10.1186/s40981-023-00676-y (PMC10673756; doi:10.1186/s40981-023-00676-y)
Supplement: Supplementary file 1 — Additional file 1: Supplementary Figure 1. CONSORT 2010 Flow Diagram. [file 40981_2023_676_MOESM1_ESM.pptx]

## Slide 1
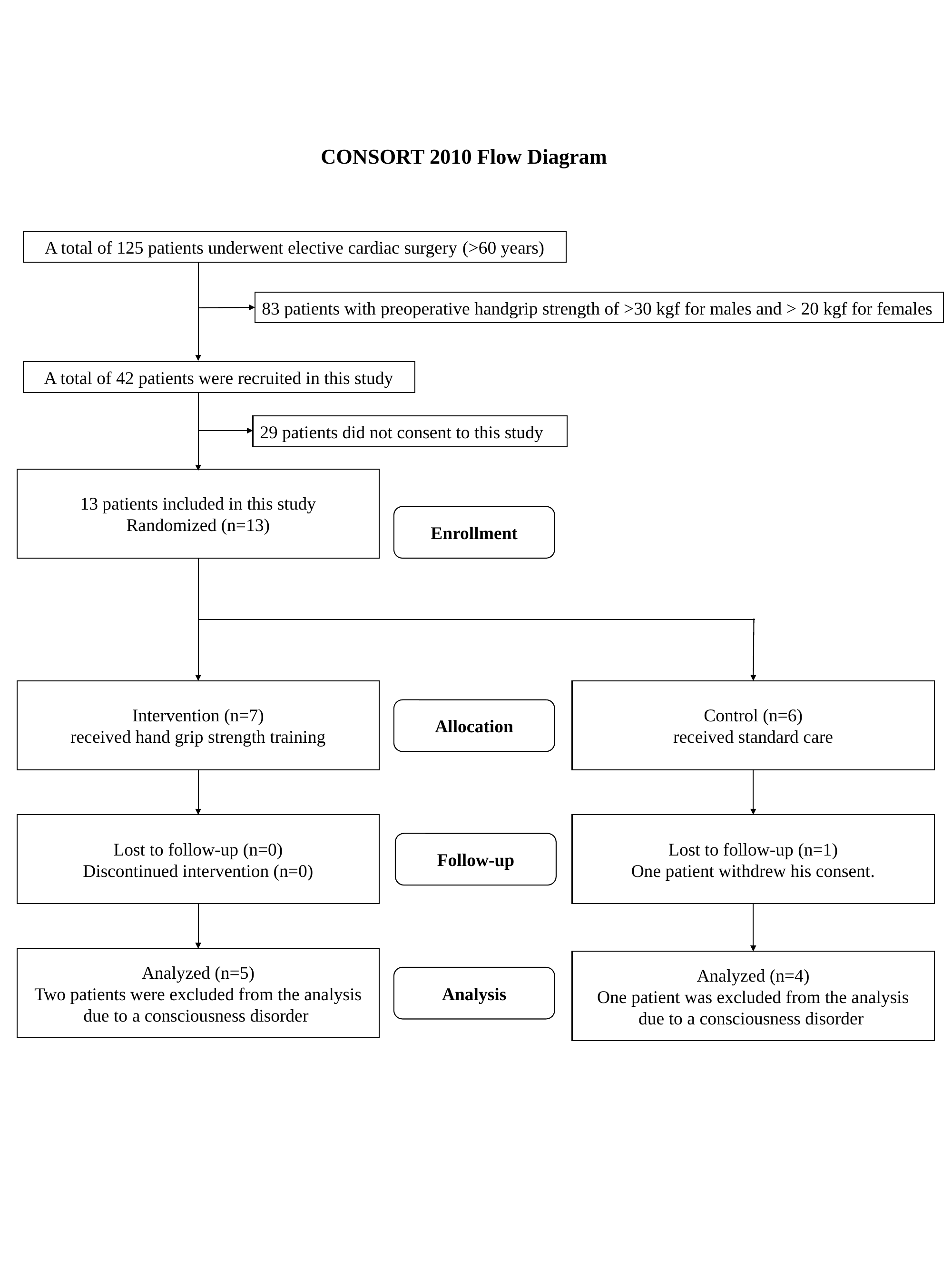

CONSORT 2010 Flow Diagram
A total of 125 patients underwent elective cardiac surgery (>60 years)
83 patients with preoperative handgrip strength of >30 kgf for males and > 20 kgf for females
A total of 42 patients were recruited in this study
29 patients did not consent to this study
13 patients included in this study
Randomized (n=13)
Enrollment
Intervention (n=7)
received hand grip strength training
Control (n=6)
received standard care
Allocation
Lost to follow-up (n=0)
Discontinued intervention (n=0)
Lost to follow-up (n=1)
One patient withdrew his consent.
Follow-up
Analyzed (n=5)
Two patients were excluded from the analysis
due to a consciousness disorder
Analyzed (n=4)
One patient was excluded from the analysis
due to a consciousness disorder
Analysis
