# Supplementary material for: Association between delirium and grip strength in ICU patients for cardiac surgery (D-GRIP study)
Source: JA Clin Rep. 2023 Nov 25;9:81. doi: 10.1186/s40981-023-00676-y (PMC10673756; doi:10.1186/s40981-023-00676-y)
Supplement: Supplementary file 2 — Additional file 2: Supplementary Table 1. a Demographic characteristics of the participants. b Intraoperative and ICU data of the participants. [file 40981_2023_676_MOESM2_ESM.docx]

**Table 1a.** Demographic characteristics of the participants

|  | Intervention  n=5 | Control  n=4 |
| --- | --- | --- |
| Age (years) | 75.0±4.0 | 75.0±1.6 |
| Sex (Female) | 2 (40) | 0 (0) |
| Body mass index (kg/m^2^) | 21.6±4.2 | 20.8±3.1 |
| Comorbidity |  |  |
| Cerebrovascular disease | 1 (20) | 2 (50) |
| Hypertension | 5 (100) | 4 (100) |
| Diabetes mellitus | 3 (60) | 2 (50) |
| Hemodialysis | 1 (20) | 1 (25) |
| Laboratory data |  |  |
| Serum albumin (g/dL) | 4.4±0.3 | 3.9±0.5 |
| Serum creatinine (g/dL) | 2.3±2.0 | 3.0±3.4 |
| BNP (pg/mL) | 608±791 | 155±93.1 |
| Ejection fraction (%) | 55.6±11.4 | 57.0±10.0 |
| Handgrip strength (dominant hand) |  |  |
| 1 month before surgery (kgf) | 23.6±5.9 | 23.5±4.7 |
| A day before surgery (kgf) | 28.8±7.0 | 25.0±6.1 |
| MMSE (points) | 28.4±1.6 | 29.5±0.5 |
| Daily use medication |  |  |
| H_2_ blockers | 1 (20) | 1 (25) |
| Beta blockers | 3 (60) | 3 (75) |
| Steroids | 0 (0) | 0 (0) |
| Benzodiazepines | 0 (0) | 0 (0) |
| Statins | 3 (60) | 3 (75) |

Data are presented as mean±SD or cases (%).

BNP: brain natriuretic peptide, MMSE: Mini-Mental State Examination, SD: standard deviation

**Table 1b.** Intraoperative and ICU data of the participants

|  | Intervention  n=5 | Control  n=4 |
| --- | --- | --- |
| Surgical procedure |  |  |
| CABG | 2 (40) | 3 (75) |
| Valvular | 0 (0) | 1 (25) |
| Combined | 3 (60) | 0 (0) |
| Anesthetic data |  |  |
| Anesthesia time (min) | 450±123 | 480±79 |
| Operation time (min) | 363±105 | 395±75 |
| Blood loss (mL) | 1688±983 | 1105±577 |
| During ICU data |  |  |
| Duration of intubation time (h) | 11.2±4.5 | 13±3.1 |
| Duration of ICU stay (h) | 71.4±30 | 63±24 |
| Number of CAM-ICU evaluations | 9.8±3.9 | 8.8±2.8 |

Data are presented as mean±SD or cases (%).

ICU: intensive care unit, CABG: coronary artery bypass grafting, CAM: confusion assessment method, SD: standard deviation
